# Hepatic arterial infusion chemotherapy plus lenvatinib with or without programmed cell death protein-1 inhibitors for advanced cholangiocarcinoma

**DOI:** 10.3389/fimmu.2023.1235724

**Published:** 2023-08-30

**Authors:** Zhanqi Wei, Yajing Wang, Boyang Wu, Ying Liu, Yaqin Wang, Zhizhong Ren, Xiaowei Yang, Qian Chen, Yuewei Zhang

**Affiliations:** ^1^ Hepatobiliary Pancreatic Center, Beijing Tsinghua Changgung Hospital, School of Clinical Medicine, Tsinghua University, Beijing, China; ^2^ School of Medicine, Tsinghua University, Beijing, China; ^3^ School of Clinical Medicine, Tsinghua University, Beijing, China; ^4^ Thorgene Co., Ltd., Beijing, China

**Keywords:** advanced cholangiocarcinoma, hepatic arterial infusion chemotherapy, lenvatinib, programmed cell death protein-1 inhibitor, efficacy, safety

## Abstract

**Background:**

New treatment strategies are needed to improve outcomes for patients with advanced cholangiocarcinoma (CCA) due to the limited efficacy of current first-line chemotherapy regimens. Although the combination of hepatic arterial infusion chemotherapy (HAIC), lenvatinib, and programmed cell death protein-1 (PD-1) inhibitors has been extensively evaluated in the treatment of advanced hepatocellular carcinoma, their roles in advanced CCA remain poorly understood. The purpose of this study is to compare the efficacy and safety of HAIC plus lenvatinib with or without PD-1 inhibitors in patients with advanced CCA.

**Methods:**

Between March 2019 to June 2022, patients diagnosed with advanced CAA who received HAIC plus lenvatinib with or without PD-1 inhibitors treatment were reviewed for eligibility. Efficacy was evaluated according to survival and tumor response, and safety was evaluated according to the incidence of adverse events (AEs).

**Results:**

Fifty-five patients with advanced CCA were included in the study, and they were divided into the HAIC+lenvatinib (LEN)+PD-1 inhibitors (PD-1i) group (n = 35) and HAIC+LEN group (n = 20). The median follow-up time was 14.0 (5–42) months. Patients in the HAIC+LEN+PD-1i group had significantly better PFS (HR = 0.390; 95% CI 0.189-0.806; p = 0.001) and OS (HR = 0.461; 95% CI 0.229-0.927; p = 0.01) than those in the HAIC+LEN group. The HAIC+LEN+PD-1i group showed a higher objective response rate and disease control rate than the HAIC+LEN group but did not find a significant difference. The incidence of grade 1-2 and grade 3-4 AEs was not significantly higher in the HAIC+LEN+PD-1i group compared to the HAIC+LEN group, whereas two patients (5.7%) in the HAIC+LEN+PD-1i group experienced grade 5 immune-mediated pneumonia.

**Conclusion:**

HAIC plus lenvatinib with PD-1 inhibitors is safe and well-tolerated, and has the potential to prolong the survival of patients with advanced CCA. The addition of PD-1 inhibitors may enhance the efficacy of HAIC and lenvatinib. Therefore, the combined therapy has the potential to become a treatment option for advanced CCA.

## Introduction

Cholangiocarcinoma (CCA), a malignant tumor originating from the intrahepatic, perihilar, or distal (extrahepatic) bile duct, accounts for about 3% of all digestive system malignancies and 2% of all cancer-related deaths worldwide (1, 2). Surgical treatment is the only curative method for CCA. However, as most patients are diagnosed with advanced CCA accompanied by multiple lesions and lymph node metastasis, although surgical treatment has made progress in recent years, the prognosis of CCA is still poor, with a high recurrence rate of up to 60% within two years, a reoperation rate of only 9%, and a 5-year survival rate of less than 5%. The median survival time for unresectable hilar CCA is about 10 months, while for intrahepatic CCA, it is as short as 6 months (2–4). The gemcitabine plus cisplatin regimen is considered the first-line chemotherapy standard for advanced or metastatic CCA (5). However, the efficacy of current first-line chemotherapy regimens is very limited (3, 5, 6). Therefore, new treatment strategies are needed to improve outcomes for patients with advanced CCA.

Hepatic arterial infusion chemotherapy (HAIC) of oxaliplatin, fluorouracil, and leucovorin (FOLFOX) is a regional chemotherapy that delivers high concentrations of chemotherapy drugs directly to the tumor site via the hepatic artery. Arterial administration of FOLFOX not only reduces toxicity and side effects, but also achieves higher drug concentrations and stronger anti-tumor effects than systemic delivery (7–9). FOLFOX-HAIC has demonstrated clinical benefits for patients with advanced CCA, including improved response rates and survival outcomes (8, 10).

Lenvatinib is a multi-target anti-angiogenic drug, showing favorable anti-tumor angiogenesis effect in a variety of solid tumors and improving T-cell infiltration in the tumor microenvironment (11–14). Lenvatinib has shown promising activity in the treatment of advanced CCA (15). Furthermore, many studies on hepatobiliary malignancies have confirmed the synergistic effect of lenvatinib and HAIC (14–17). Programmed cell death protein-1 (PD-1) inhibitors are a type of immunotherapy that blocks the PD-1 pathway, which is responsible for inhibiting the immune response against tumors. PD-1 inhibitors have also shown durable responses and improved survival outcomes in advanced CCA (18). However, the response rates of CCA are still unsatisfactory, whether treated with PD-1 inhibitors alone or in combination with targeted therapy.

The combination of HAIC, lenvatinib, and PD-1 inhibitors may result in synergistic anti-tumor effects, as each therapeutic approach targets different pathways involved in tumor growth and survival, and concurrent use of multiple treatments can overcome drug resistance which often limits clinical efficacy of single-agent therapies (19–21). Although the combination of HAIC, lenvatinib and PD-1 inhibitors has been extensively evaluated in the treatment of advanced hepatocellular carcinoma, their roles in advanced CCA remain poorly understood. Thus, we hypothesize that this is a promising combinatorial approach for advanced CCA. The objective of this study is to evaluate the safety and efficacy of HAIC plus lenvatinib with or without PD-1 inhibitors in patients with advanced CCA, and to compare the therapeutic effect of these two treatment regimens. The findings from this study may provide new insights into the treatment of advanced CCA and guide the development of future therapeutic strategies.

## Materials and methods

After the institutional review board (IRB) of Beijing Tsinghua Changgung Hospital reviewed and approved the patient data analysis, medical records of patients with advanced CCA from our center who were received HAIC plus lenvatinib with or without PD-1 inhibitors at our center from March 2019 to June 2022 were reviewed. The study was conducted ethically, following the guidelines of the Declaration of Helsinki, and all participants provided informed consent for treatment.

### Patient selection

Patient inclusion criteria for this study were as follows (1): Patients aged from 18 to 80 years (2); Patients diagnosed with advanced CCA through enhanced computed Tomography (CT), magnetic resonance imaging (MRI), and histopathology at our center (3); Patients with advanced CCA with multiple lesions, vascular invasion, local lymph node metastasis, and distant metastasis (4); Patients who opted out of systemic chemotherapy (5); Patients with an Eastern Cooperative Oncology Group performance status (ECOG PS) within the range of 0-2 prior to HAIC treatment (6); Patients who underwent at least one cycle of HAIC plus lenvatinib with or without two cycles of PD-1 inhibitor treatment (7); Patients with complete follow-up records.

In contrast, exclusion criteria were (1): Patients with co-existing malignant tumors (2); Patients with histopathologically confirmed mixed tumors of hepatocellular carcinoma and CCA (3); Patients with histopathologically confirmed gallbladder carcinoma (3); Patients who were allergic to chemotherapy drugs, lenvatinib, or PD-1 inhibitors (4); Patients without enhanced CT or MRI images before treatment (5); Patients with uncontrolled hypertension and severe cardiovascular disease (6); Patients classified as Child-Pugh class C (7); Patients with a history of active autoimmune diseases (8); Patients with a history of idiopathic pneumonia and drug-induced pneumonia (9); Patients with severe active infections excluding hepatitis B virus (HBV) and hepatitis C virus (HCV) (10); Patients with severe gastrointestinal bleeding within 4 weeks prior to treatment (11); Patients with severe thrombosis within 6 months prior to treatment (12); Patients who received other treatments, including but not limited to transarterial chemoembolization (TACE), radiotherapy, or systemic chemotherapy (13); Patients who previously participated in clinical trials (14); Patients missing clinical data or follow-up.

**Figure 1:**
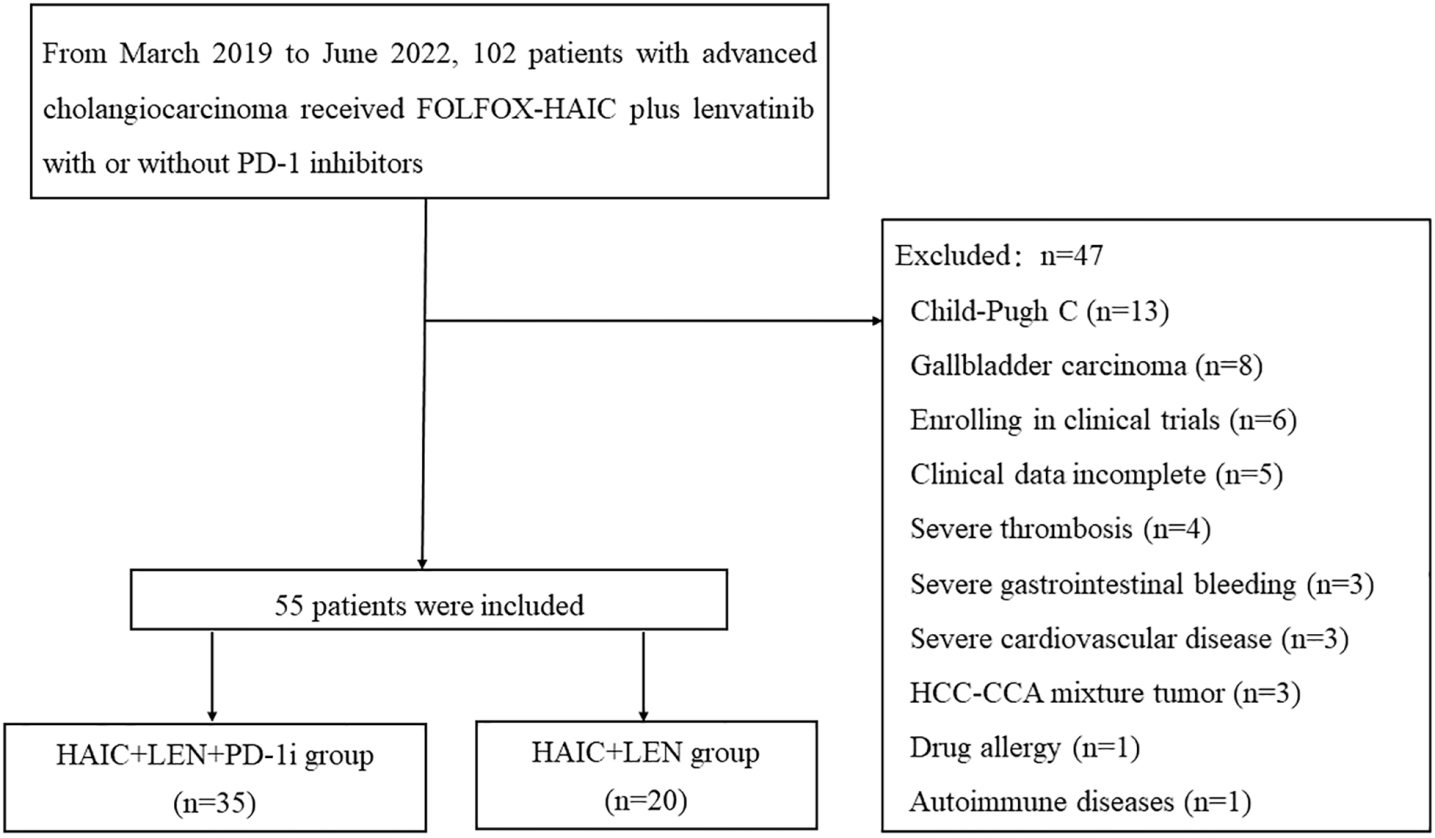
Flow diagram of study design. FOLFOX-HAIC, hepatic arterial infusion chemotherapy of oxaliplatin, fluorouracil, and leucovorin; HCC, hepatocellular carcinoma; CCA, cholangiocarcinoma; LEN, lenvatinib; PD-1i, PD-1 inhibitors.

Prior to initial treatment, laboratory data and enhanced CT or MRI images were collected and documented within 2 weeks. The inclusion and exclusion process of this study was depicted in **Figure 1**, leading to the final inclusion of 55 patients.

### Data collection

We sourced clinical data from the electronic medical record system of Beijing Tsinghua Changgung Hospital. The following parameters were collected and analyzed for the study: Age, gender, number of HAIC treatment cycles, comorbidities, HBV status, ECOG-PS score, number of tumors, white blood cell count (WBC), neutrophil count (NEUT), lymphocyte count (LY), platelet count (PLT), serum albumin (ALB), aspartate aminotransferase (AST), alanine aminotransferase (ALT), total bilirubin count (TBIL), alkaline phosphatase (ALP), gamma-glutamyl transferase (GGT), cholinesterase (CHE), liver function classification (Child-Pugh score), carcinoembryonic antigen (CEA), carbohydrate antigen 19-9 (CA19-9), protein induced by vitamin K absence II (PIVKA-II), tumor stage (TNM staging based on the American Joint Committee on Cancer 8th edition), presence of portal vein tumor thrombus, vascular invasion, distant metastases, and treatment with pre-emptive percutaneous transhepatic cholangial drainage (PTCD).

### Treatment protocol

Patients received liver protection, antiemetic, pain relief and other symptomatic treatments prior to HAIC. For patients with hyperbilirubinemia, PTCD drainage was performed before surgery and HAIC was administered after bilirubin levels had decreased to less than three times the normal range. Each HAIC treatment cycle lasts between 1 to 3 days, during which femoral artery puncture and catheter insertion are performed with the aid of digital subtraction angiography (DSA) to accurately select tumor feeding arteries. If necessary, the gastroduodenal artery or right gastric artery can be embolized with a spring coil. 5-fluorouracil is continuously administered for up to 12 hours per day at a total dose of 1500mg, while patients receive 50mg of oxaliplatin and 300mg of calcium folinate nightly for two hours. The interval between two HAIC treatment cycles is anywhere from 2 to 5 weeks, and patients receive 1 to 9 cycles of HAIC treatment.

Prior to HAIC treatment or one week after treatment, depending on bilirubin levels, patients were given lenvatinib (Japan Eisai Co, Ltd) orally at a dosage of either 8 mg (≤60 kg) or 12 mg (>60 kg) depending on their body weight. If patients were unable to tolerate HAIC, they continued taking lenvatinib; if patients were unable to tolerate lenvatinib, they reduced or stopped taking lenvatinib and continued with HAIC treatment.

PD-1 inhibitors were administered via intravenous drip within 1 to 2 weeks after HAIC treatment, with a dose of 200 mg every 3 weeks. Patients must receive at least 2 cycles of PD-1 inhibitor treatment. For patients with hyperbilirubinemia, the treatment can be initiated once the bilirubin level returns to within 2 times the normal range after PTCD drainage. The PD-1 inhibitors used in this study mainly include camrelizumab (28/35, 80.0%), sintilimab (4/35, 11.4%), and tislelizumab (3/35, 8.6%).

Treatment was discontinued in the event of disease progression (PD), the patient being unable to tolerate toxic or adverse reactions, patient refusal of treatment or change of treatment regimen. Enhanced computed tomography (CT) or magnetic resonance imaging (MRI) was performed every 2 to 3 months, while follow-up visits were scheduled every 3 months.

### Outcomes and assessments

The primary endpoints were overall survival (OS) and progression-free survival (PFS). OS was defined as the duration from the commencement of the initial therapy till the occurrence of death owing to any cause or the last follow-up. PFS was the duration from the beginning of the primary therapy till either the progression of the disease or the administration of bridging therapy and transplantation, or the last follow-up. Modified Response Evaluation Criteria in Solid Tumors (mRECIST) and RECIST version 1.1 were the standard methods employed by radiologists and hepatobiliary surgeons to assess the tumor response. The response criteria involved the determination of complete response (CR), partial response (PR), stable disease (SD), and PD. Objective response rate (ORR) was defined as the sum of CR and PR, whereas disease control rate (DCR) was determined from the sum of CR, PR, and SD. The National Cancer Institute Common Terminology Criteria for Adverse Events (CTCAE) version 5.0 was utilized to evaluate treatment-related adverse events (AEs).

### Statistical analysis

To compare the baseline characteristics of patients receiving HAIC plus lenvatinib with PD-1 inhibitors and HAIC plus lenvatinib without PD-1 inhibitors for advanced CCA, statistical tests including Pearson’s chi-square test, Fisher’s exact test, or Wilcoxon rank sum test were conducted. Normally distributed variables were described using mean ± standard error (SE), while median was used for non-normally distributed variables. Survival analysis was performed using the Kaplan-Meier method, and differences in survival curves were assessed using the log-rank test. Baseline variables that were deemed clinically relevant or demonstrated a univariate association with outcome were included in a multivariate Cox proportional-hazards regression model. The variables were selected judiciously, taking into account the number of available events, to ensure that the final model was parsimonious. This regression analysis comprised patients’ basic information, treatment method, tumor status, and more to calculate hazard ratios (HR) and confidence intervals (CI). R4.2.2 software was used for all descriptive and multivariate analyses. Statistical significance was considered when *p*-values <0.05 using a two-tailed test. All data analysis was conducted using R language and Statistical Package for the Social Sciences (SPSS, version 24.0, IBM, USA).

## Results

### Patient and tumor characteristics

From March 2019 to June 2022, a total of 55 advanced CCA patients were enrolled in this study. 35 patients received HAIC plus lenvatinib with PD-1 inhibitors, while 20 patients received only HAIC plus lenvatinib treatment. The HAIC+LEN+PD-1i group received 1 to 6 cycles of HAIC, with a median of 4 cycles, while the HAIC+LEN group received 1 to 9 cycles of HAIC, with a median of 3 cycles. **Table 1** displayed the demographic data and baseline characteristics of the two groups, which did not differ significantly in clinical variables.

**Table 1:** Demographics of patients included in the study.

Characteristic	HAIC+LEN+PD-1i	HAIC+LEN	*p*-value
	(n=35)	(n=20)	
Patient characteristics			
Age, mean ± SD	59.543 ± 11.76	59.8 ± 9.28	0.933
Sex, n (%)			0.431
Female	12 (34.3%)	9 (45.0%)	
Male	23 (65.7%)	11 (55.0%)	
Hepatitis, n (%)			0.696
Negative	27 (77.1%)	15 (75.0%)	
HBV	7 (20.0%)	5 (25.0%)	
HCV	1 (2.9%)	0 (0.0%)	
Hypertension, n (%)	12 (34.3%)	9 (45.0%)	0.431
Diabetes mellitus, n (%)	5 (14.3%)	3 (15.0%)	0.999
Coronary artery disease, n (%)	2 (5.7%)	1 (5.0%)	0.999
Child-Pugh, n (%)			0.168
A	16 (45.7%)	13 (65.0%)	
B	19 (54.3%)	7 (35.0%)	
HAIC cycle, median (IQR)	4 (2, 5)	3 (2.75, 4)	0.471
Tumor characteristics			
Size of largest nodule, mm	57.926 ± 33.459	64.111 ± 31.994	0.54
Vascular invasion, n (%)	27 (77.1%)	14 (70.0%)	0.559
Lymph node metastasis, n (%)	19 (54.3%)	14 (70.0%)	0.252
Extrahepatic metastasis, n (%)	12 (34.3%)	9 (45.0%)	0.431
Thrombus, n (%)			0.227
Absent	20 (57.1%)	9 (45.0%)	
Branch of portal vein	10 (28.6%)	10 (50.0%)	
Main portal vein	5 (14.3%)	1 (5.0%)	
Laboratory test characteristics			
WBC, median (IQR), ×10^9^/L	6.13 (4.225, 7.855)	5.895 (4.725, 8.545)	0.421
NEUT, median (IQR), ×10^9^/L	3.89 (2.8, 5.44)	3.61 (2.9925, 7.14)	0.564
LY, median (IQR), ×10^9^/L	1.06 (0.725, 1.675)	1.605 (1.1275, 1.85)	0.126
Hb, mean ± SD, g/L	118.37 ± 20.783	118.95 ± 20.096	0.920
PLT, median (IQR), ×10^9^/L	181 (128.5, 221.5)	232 (127.25, 265)	0.336
ALB, mean ± SD, g/L	36.323 ± 4.1009	35.86 ± 5.0638	0.713
AST, median (IQR), U/L	39.7 (25.7, 59.75)	31 (23.25, 48.925)	0.372
ALT, median (IQR), U/L	34.8 (22.8, 72.85)	30.8 (19.95, 39.575)	0.563
ALP, median (IQR), U/L	155 (118.5, 263)	182 (95.75, 292)	0.937
GGT, median (IQR), U/L	193 (74.5, 237.5)	114.5 (72.25, 318.5)	0.726
CHE, median (IQR), U/L	4692 (3363, 6345.5)	5221.5 (3257, 6996.5)	0.882
TBIL, median (IQR), μmol/L	24.4 (12.25, 59.9)	20.35 (13, 43.675)	0.713
AFP, median (IQR), ng/ml	3.02 (2.03, 3.86)	3.12 (2.44, 9.0275)	0.399
CEA, median (IQR), μg/L	3.49 (1.96, 5.2)	3.11 (2.0525, 7.6175)	0.951
CA19-9, median (IQR), U/ml	40.71 (8.925, 666.48)	168 (62.245, 1472.9)	0.069
PVIK-II, median (IQR), mAU/ml	27.77 (20.89, 150.11)	25.03 (19.035, 142.25)	0.773

HAIC, hepatic artery infusion chemotherapy; LEN, lenvatinib; PD-1i, programmed cell death protein-1 inhibitors; SD, standard deviation; HBV, hepatitis B virus; HCV, hepatitis C virus; TNM, tumor-node-metastasis; IQR, interquartile range; WBC, white blood cell; NEUT, neutrophil; LY, lymphocyte; Hb, hemoglobin; PLT, blood platelet; ALB, albumin; AST, aspartate aminotransferase; ALT, alanine aminotransferase; ALP, alkaline phosphatase; GGT, gamma-glutamyl transferase; CHE, cholinesterase; TBIL, total bilirubin; AFP, alpha-Fetoprotein; CEA, carcinoembryonic antigen; CA19-9, carbohydrate antigen 19-9; PIVKA-II, protein induced by vitamin K absence II.

### Survival

**Figure 2:**
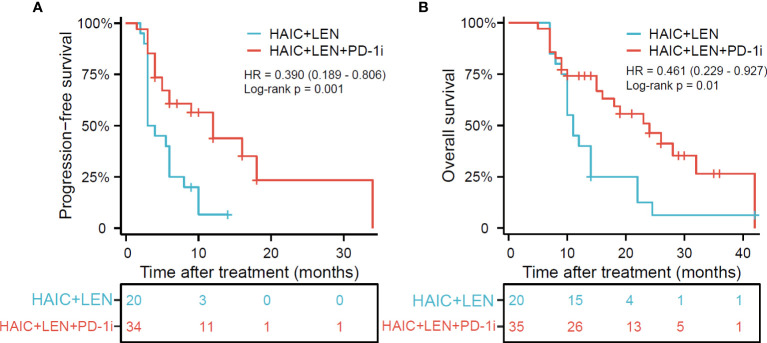
Kaplan-Meier plot for progression free survival **(A)** and overall survival **(B)** based on the HAIC+LEN+PD-1i group compared with the HAIC+LEN group. HAIC, hepatic arterial infusion chemotherapy; LEN, lenvatinib; PD-1i, PD-1 inhibitors; OS, overall survival; PFS, progression free survival. * One patient in the HAIC+LEN+PD-1i group was unable to accurately assess the time of disease progression, therefore only 34 patients were included in the evaluation of progression-free survival.

The median follow-up time was 14.0 months (range, 5-42 months), with the last follow-up date being April 10, 2023. As shown in **Figure 2**, patients in the HAIC+LEN+PD-1i group had significantly better PFS (HR = 0.390; 95% CI 0.189-0.806; *p* = 0.001; **Figure 2A**) and OS (HR = 0.461; 95% CI 0.229-0.927; *p* = 0.01; **Figure 2B**) than those in the HAIC+LEN group. The median OS of the HAIC+LEN+PD-1i and HAIC+LEN groups were 16 months and 11 months, respectively (*p* = 0.138), while the median PFS of the two groups were 6.5 months and 3.5 months, respectively (*p* = 0.020). Notably, the 1-year and 2-year OS rates of the HAIC+LEN+PD-1i group were significantly higher than those of the HAIC+LEN group (*p* = 0.012 and *p*= 0.004, respectively), and the 3-month, 6-month, 1-year, and 2-year PFS rates of the HAIC+LEN+PD-1i group were significantly higher than those of the HAIC+LEN group (*p* = 0.004, *p* = 0.007, *p* = 0.001, and *p* = 0.004, respectively) (**Table 2**).

**Table 2:** The overall survival and progression-free survival rates between the two groups.

	HAIC+LEN+PD-1i	HAIC+LEN	*p*-value
	(n=35/34)	(n=20)	
PFS, median (IQR), months	6.5 (4, 11.5)	3.5 (3, 6.5)	0.020*
OS, median (IQR), months	16 (9.5, 25)	11 (9.75, 14)	0.138
OS rates, n (%)			
3-month	35/35 (100%)	20/20 (100%)	0.999
6-month	34/35 (97.1%)	20/20 (100%)	0.999
1-year	26/35 (74.3%)	8/20 (40.0%)	0.012*
2-year	19/35 (54.3%)	3/20 (15.0%)	0.004*
PFS rates, n (%)			
3-month	30/34 (88.2%)	10/20 (50.0%)	0.004*
6-month	22/34 (64.7%)	5/20 (25.0%)	0.007*
1-year	19/34 (55.9%)	2/20 (10.0%)	0.001*
2-year	17/34 (50.0%)	2/20 (10.0%)	0.004*

HAIC, hepatic artery infusion chemotherapy; LEN, lenvatinib; PD-1i, programmed cell death protein-1 inhibitors; OS, overall survival; PFS, progression-free survival.

*Denotes a p-value <0.05.

**Figure 3:**
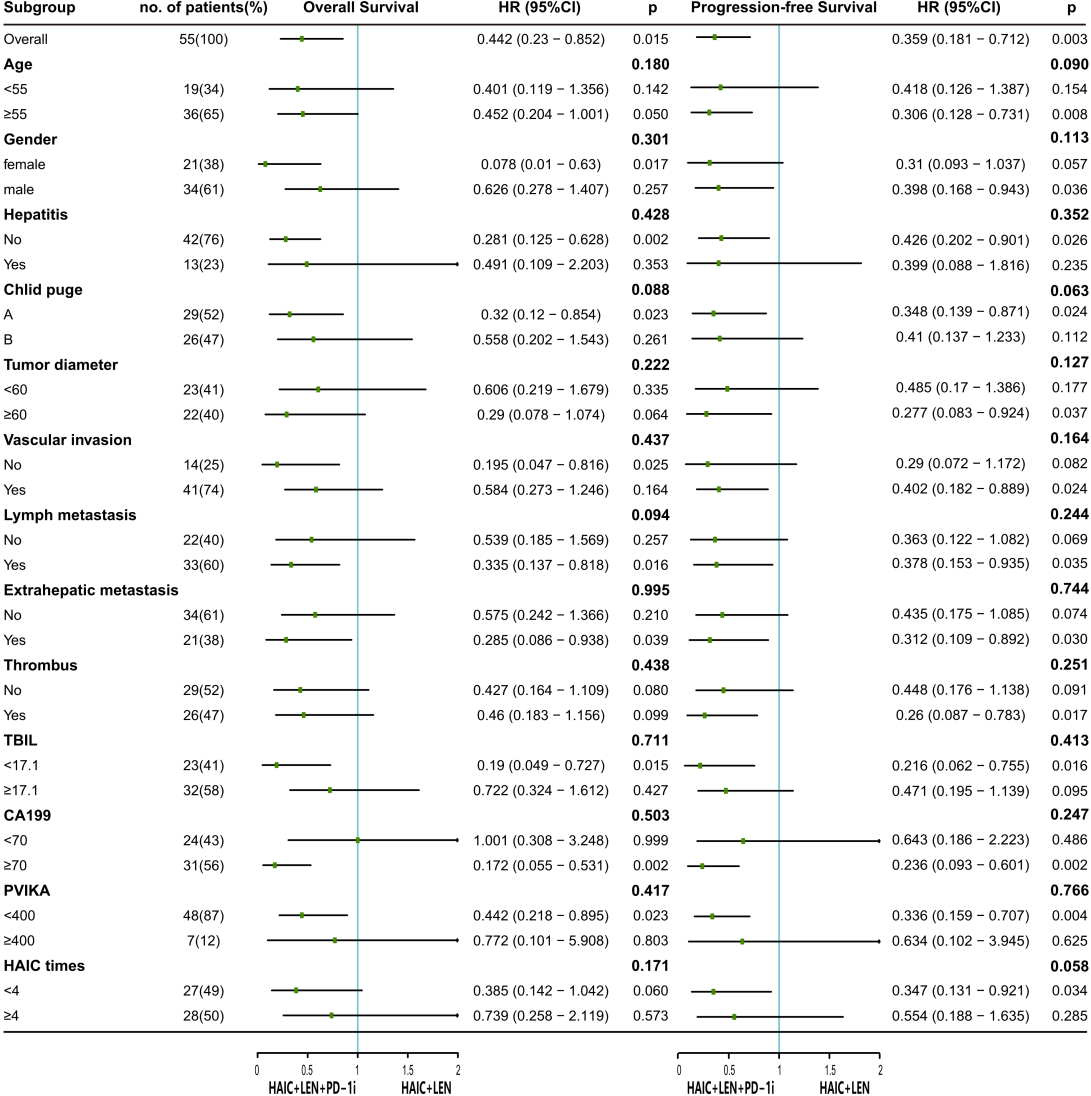
The forest plot analysis of factors associated with overall survival and progression free survival. HAIC, hepatic arterial infusion chemotherapy; LEN, lenvatinib; PD-1i, PD-1 inhibitors; HR, hazard ratio; 95% CI, 95% confidence interval; TBIL, total bilirubin; CA199, carbohydrate antigen 199; PVIK-II, protein induced by vitamin K absence II.

As shown in **Figure 3**, HAIC+LEN+PD-1i provided a clinical benefit for OS in patients with lymph metastasis (HR = 0.335; 95% CI 0.137-0.818; *p* = 0.016), extrahepatic metastasis (HR = 0.285; 95% CI 0.086-0.938; *p* = 0.039), and high CA199 level (HR = 0.172; 95% CI 0.055-0.531; *p* = 0.002), and provided a clinical benefit for PFS in patients with large tumor (HR = 0.277; 95% CI 0.083-0.924; *p* = 0.037), vascular invasion (HR = 0.402; 95% CI 0.182-0.889; *p* = 0.024), lymph metastasis (HR = 0.378; 95% CI 0.153-0.935; *p* = 0.035), extrahepatic metastasis (HR = 0.312; 95% CI 0.109-0.892; *p* = 0.030), portal vein tumor thrombus (HR = 0.26; 95% CI 0.087-0.783; *p* = 0.017), and high CA199 level (HR = 0.236; 95% CI 0.093-0.601; *p* = 0.002).

### Tumor response

Radiographic evaluation by RECIST version 1.1, at the 3-month post-treatment imaging assessment, demonstrated that 7 (20.0%) patients in the HAIC+LEN+PD-1i group had PD, 18 (51.4%) patients showed SD, 9 (25.7%) patients achieved PR, and 1 (2.9%) patient achieved CR, resulting in an ORR of 28.6% and DCR of 80.0%. In the HAIC+LEN group, 7 (35.0%) patients had PD, 9 (45.0%) patients had SD, and 4 (20.0%) patients achieved PR, resulting in an ORR of 20.0% and DCR of 65.0%. The HAIC+LEN+PD-1i group showed a higher ORR and DCR than the HAIC+LEN group but did not find a significant difference (**Table 3**).

**Table 3:** Tumor response rates and operation rates between the two groups.

	HAIC+LEN+PD-1i	HAIC+LEN	*p*-value
	(n=35)	(n=20)	
Tumor response, n (%)			
CR	1 (2.9%)	0 (0.0%)	0.999
PR	9 (25.7%)	4 (20.0%)	0.881
SD	18 (51.4%)	9 (45.0%)	0.646
PD	7 (20.0%)	7 (35.0%)	0.219
ORR	10 (28.6%)	4 (20.0%)	0.483
DCR	80.0%	13 (65.0%)	0.219
Operation, n (%)			
Surgical resection	4 (11.4%)	1 (5.0%)	0.756
Liver transplantation	2 (5.7%)	1 (5.0%)	0.999

HAIC, hepatic artery infusion chemotherapy; LEN, lenvatinib; PD-1i, programmed cell death protein-1 inhibitors; CR, complete response; PR, partial response; SD, stable disease; PD, progressive disease; ORR, objective response rate; DCR, disease control rate.

In the HAIC+LEN+PD-1i group, 4 patients underwent surgical resection, of which 3 patients were pathologically confirmed complete necrosis of the tumor, 2 patients received liver transplantation, of which 1 patient was pathologically confirmed 90% necrosis, and 1 patient was pathologically confirmed 30% necrosis. In the HAIC group, 1 patient underwent surgical resection, and 1 patient underwent liver transplantation. All of them died of disease progression or recurrence. The surgical conversion rates of two groups were 17.1% and 10%, respectively (**Table 3**).

### Safety and tolerability

As shown in **Table 4**, based on the CTCAE 5.0 standards, the incidence of AEs for the HAIC+LEN+PD-1i group and the HAIC+LEN group were 97.1% and 90%, respectively. In the HAIC+LEN+PD-1i group, nausea (77.1%), fatigue (71.4%), and vomiting (65.7%) were the most common grade 1-2 AEs, and the most common grade 3-4 AEs were hypertension (42.9%), hand-foot syndrome (8.6%), and immune-mediated pneumonia (8.6%). In the HAIC+LEN group, the most common grade 1-2 AEs were fatigue (70.0%), vomiting (65.0%), and elevated transaminases (65.0%), and the most common grade 3-4 AEs were hypertension (45.0%), and hand-foot syndrome (20.0%). There was no significant difference observed in the incidence of grade 1-2 and grade 3-4 AEs between the two groups. However, two patients (5.7%) in the HAIC+LEN+PD-1i group experienced grade 5 immune-mediated pneumonia, and both died due to the grade 5 AE induced by treatment.

**Table 4:** The adverse events in the two groups according to Common Terminology Criteria for Adverse Events version 5.0.

	Grade 1-2 AEs			Grade 3-4 AEs		
n (%)	HAIC+LEN+PD-1i (n=35)	HAIC+LEN (n=20)	*p*-value	HAIC+LEN+PD-1i (n=35)	HAIC+LEN (n=20)	*p*-value
Nausea	27 (77.1%)	12 (60.0%)	0.178	0 (0%)	0 (0%)	/
Vomiting	23 (65.7%)	13 (65.0%)	0.957	0 (0%)	0 (0%)	/
Abdominal pain	18 (51.4%)	9 (45.0%)	0.646	0 (0%)	1 (5.0%)	0.364
Abdominal distention	9 (25.7%)	6 (30.0%)	0.731	0 (0%)	0 (0%)	/
Diarrhea	6 (17.1%)	2 (10.0%)	0.696	1 (2.9%)	1 (5.0%)	0.999
Fever	12 (34.3%)	9 (45.0%)	0.431	2 (5.7%)	0 (0%)	0.529
Hypertension	14 (40.0%)	9 (45.0%)	0.718	15 (42.9%)	9 (45.0%)	0.877
Hand-foot syndrome	7 (20.0%)	3 (15.0%)	0.731	3 (8.6%)	4 (20.0%)	0.242
Gastric mucosal bleeding	1 (2.9%)	2 (10.0%)	0.546	2 (5.7%)	1 (5.0%)	0.999
Joint pain	1 (2.9%)	3 (15.0%)	0.131	0 (0%)	0 (0%)	/
Fatigue	25 (71.4%)	14 (70.0%)	0.911	2 (5.7%)	1 (5.0%)	0.999
Infection	5 (14.3%)	1 (5.0%)	0.399	1 (2.9%)	1 (5.0%)	0.999
Thrombocytopenia	17 (48.6%)	10 (50.0%)	0.919	2 (5.7%)	1 (5.0%)	0.999
Leukopenia	21 (60.0%)	8 (40.0%)	0.153	2 (5.7%)	1 (5.0%)	0.999
Elevated transaminases	20 (57.1%)	13 (65.0%)	0.567	1 (2.9%)	0 (0%)	0.999
Elevated bilirubin	6 (17.1%)	5 (25.0%)	0.483	2 (5.7%)	0 (0%)	0.529
Immune-mediated pneumonia	0 (0%)	0 (0%)	/	3 (8.6%)	0 (0%)	0.293
Hypothyroidism	2 (5.7%)	0 (0%)	0.529	1 (2.9%)	0 (0%)	0.999
Immune-mediated myocarditis	0 (0%)	0 (0%)	/	1 (2.9%)	0 (0%)	0.999

AEs, adverse events; HAIC, hepatic artery infusion chemotherapy; LEN, lenvatinib; PD-1i, programmed cell death protein-1 inhibitors.

### Prognostic factor analysis

The prognostic factors for survival are shown in **Tables 5**, **6**. The presence of PD-1 inhibitors was identified as an independent protective factor for PFS (HR = 0.368; 95% CI 0.168-0.807; *p* = 0.013). In addition, multivariate analyses identified that lymph metastasis was a risk factor for OS (HR = 2.393; 95% CI 1.076-5.326; *p* = 0.032), and extrahepatic metastasis was a risk factors for PFS (HR = 2.592; 95% CI 1.155-5.814; *p* = 0.021), while Child-pugh grade A was a protective factor for PFS (HR = 0.428; 95% CI 0.187-0.978; *p* = 0.044).

**Table 5:** Univariate and multivariate analysis of risk factors for overall survival.

Characteristic	Univariate analysis			Multivariate analysis		
	HR	95% CI	*p*-value	HR	95% CI	*p*-value
Age, <55 vs. ≥55 (years)	0.557	0.197 - 1.574	0.269			
Sex, female vs. male	0.78	0.195 - 3.123	0.726			
HAIC cycles, <4 vs. ≥4	1.464	0.56 - 3.831	0.437			
Hepatitis, present vs. absent	0.513	0.174 - 1.51	0.225			
Child-pugh grade, A vs. B	0.639	0.165 - 2.474	0.517			
Tumor diameter, <60 vs. ≥60 (mm)	1.765	0.508 - 6.129	0.371			
Vasclar invasion, present vs. absent	1.136	0.391 - 3.297	0.815			
Lymph metastasis, present vs. absent	1.408	0.43 - 4.613	0.572	2.393	1.076 - 5.326	0.032*
Extrahepatic metastasis, present vs. absent	0.346	0.025 - 4.786	0.429	0.227	0.029 - 1.775	0.158
Portal vein tumor thrombus, present vs. absent	2.435	0.676 - 8.763	0.173			
Main tumor thrombus, present vs. absent	1.559	0.222 - 10.972	0.656			
Branch tumor thrombus, present vs. absent	0.598	0.236 - 1.515	0.278			
TBIL, <17.1 vs. ≥17.1 (μmol/L)	0.828	0.223 - 3.082	0.779			
CA199, <70 vs. ≥70 (U/mL)	2.882	1.028 - 8.082	0.044*			
PVIK-II, <400 vs. ≥400 (mU/mL)	/	/	0.603	/	/	0.012*
PD-1i treatment, present vs. absent	0.615	0.234 - 1.617	0.324			

HR, hazard ratio; 95% CI, 95% confidence interval; HAIC, hepatic artery infusion chemotherapy; TBIL, total bilirubin; CA199, carbohydrate antigen 199; PVIK-II, protein induced by vitamin K absence II; PD-1i, programmed cell death protein-1 inhibitors.

*Denotes a p-value <0.05.

**Table 6:** Univariate and multivariate analysis of risk factors for progression-free survival.

Characteristic	Univariate analysis			Multivariate analysis		
	HR	95% CI	*p*-value	HR	95% CI	*p*-value
Age, <55 vs. ≥55 (years)	0.905	0.33 - 2.476	0.845			
Sex, female vs. male	1.45	0.536 - 3.921	0.465			
HAIC cycles, <4 vs. ≥4	0.566	0.205 - 1.563	0.272			
Hepatitis, present vs. absent	0.605	0.151 - 2.417	0.477			
Child-pugh grade, A vs. B	0.433	0.108 - 1.728	0.236	0.428	0.187 - 0.978	0.044*
Tumor diameter, <60 vs. ≥60 (mm)	0.815	0.282 - 2.359	0.707			
Vascular invasion, present vs. absent	1.522	0.422 - 5.495	0.521			
Lymph metastasis, present vs. absent	0.685	0.184 - 2.547	0.572			
Extrahepatic metastasis, present vs. absent	2.746	1.001 - 7.529	0.05	2.592	1.155 - 5.814	0.021*
Portal vein tumor thrombus, present vs. absent	/	/	0.74			
Main tumor thrombus, present vs. absent	0.646	0.214 - 1.948	0.438			
Branch tumor thrombus, present vs. absent	/	/	0.984			
TBIL, <17.1 vs. ≥17.1 (μmol/L)	1.28	0.427 - 3.84	0.66			
CA199, <70 vs. ≥70 (U/mL)	2.585	0.638 - 10.468	0.183			
PVIK-II, <400 vs. ≥400 (mU/mL)	0.521	0.087 - 3.12	0.475			
PD-1i treatment, present vs. absent	0.283	0.106 - 0.757	0.012*	0.368	0.168 - 0.807	0.013*

HR, hazard ratio; 95% CI, 95% confidence interval; HAIC, hepatic artery infusion chemotherapy; TBIL, total bilirubin; CA199, carbohydrate antigen 199; PVIK-II, protein induced by vitamin K absence II; PD-1i, programmed cell death protein-1 inhibitors.

*Denotes a p-value <0.05.

## Discussion

This is the first clinical study to compare the efficacy and safety of HAIC plus levatinib with or without PD-1 inhibitors in the treatment of advanced CCA. It represented a potential shift in the approach to improving local chemotherapy response in advanced CCA patients. We found that the combination of HAIC plus lenvatinib with PD-1 inhibitors was promising, with significantly better OS and PFS compared to patients who received HAIC plus lenvatinib without PD-1 inhibitors. It is important to note that the incorporation of PD-1 inhibitors for advanced CCA patients did not result in a higher occurrence of AEs, and effective interventions were utilized to mitigate most of AEs observed in both groups. Furthermore, the study identified lymph metastasis, extrahepatic metastasis, and Child-pugh grade B as predictors of unfavorable prognosis.

For advanced CCA, there are several local treatment options available, such as radiofrequency ablation, radiotherapy, transarterial chemoembolization (TACE), and HAIC. Among these options, HAIC appears to have a unique advantage in treating CCA, as it can target diffuse and multiple lesions regardless of tumor size, location, or associated hyperbilirubinemia (8, 9, 16, 22–24). The ABC-06 clinical trial demonstrated that advanced CCA patients who had failed first-line chemotherapy could benefit from FOLFOX chemotherapy (25). FOLFOX-HAIC not only reduces systemic toxicity and side effects, but also achieves higher local drug concentrations and stronger anti-tumor effects than systemic delivery (7–9). A prospective phase II study reported a total effective rate of 67.6%, DCR of 89.2%, median PFS of 12.2 months, and median OS of 20.5 months with FOLFOX-HAIC treatment in advanced perihilar CCA (8). Ishii et al. (10) found that advanced intrahepatic CCA patients treated with FOLFOX-HAIC had significantly better OS than those receiving standard chemotherapy. Therefore, FOLFOX-HAIC may be an encouraging treatment option for advanced CCA due to its high tumor control, survival benefit, and low toxicity.

Lenvatinib is a small molecule tyrosine kinase inhibitor that targets vascular endothelial growth factor receptor (VEGFR) 1-3, fibroblast growth factor receptor (FGFR) 1-4, and platelet-derived growth factor receptor (PDGFR)-α, thereby inhibiting tumor angiogenesis, and has shown some efficacy against various solid tumors (13). These receptors are also highly expressed in CCA. Therefore, lenvatinib may be a potential systemic targeted therapy for CCA and has been used as second-line therapy for CCA (4, 26). Lenvatinib has demonstrated anti-tumor activity in CCA and has a tolerable safety profile. A multicenter phase II study reported by Ueno et al. showed that median PFS was 3.19 months, while median OS was 7.35 months in unresectable CCA patients who received lenvatinib treatment (15). While direct comparisons are not feasible when considering differences in background characteristics, the PFS and OS demonstrated in both groups of patients in our study were superior to those achieved with lenvatinib monotherapy for unresectable CCA. This indirectly confirms the safety and efficacy of the combination therapy.

Immunotherapy has shown promising results in various malignancies, including advanced biliary tract tumors treated with PD-1 inhibitors. Deng et al. conducted a retrospective analysis of 42 advanced intrahepatic CCA patients treated with PD-1 inhibitors and reported a median OS, PFS, and time to progression (TTP) of 19.3, 11.6, and 11.6 months, respectively (18). To improve the clinical efficacy of PD-1 inhibitors for advanced CCA, various combination therapies are being explored, such as PD-1 inhibitors combined with chemotherapy, targeted therapy, and local treatment (27, 28). The phase III TOPAZ-1 study reported by Oh et al. (29) demonstrated that Gemcitabine and cisplatin plus immunotherapy showed promising efficacy and acceptable safety in patients with biliary tract cancer. Recently, durvalumab combined with gemcitabine and cisplatin has been confirmed as a first-line treatment by the guidelines of Food and Drug Administration (FDA) and National Comprehensive Cancer Network (NCCN). It is worth noting that the PFS in our study was shorter compared to the PFS in the TOPAZ-1 study (3.5 months vs 5.7 months). We believe that this difference may be attributed to the retrospective nature of our study, where the inclusion criteria were more lenient compared to the TOPAZ-1 study, resulting in patients with poorer baseline characteristics, such as older age and more severe underlying disease. These factors could potentially impact the patients’ survival. Furthermore, this may reflect a reality that HAIC is a localized treatment method primarily targeting liver disease. In cases of extrahepatic disease, the effectiveness of HAIC in achieving systemic control may be suboptimal compared to conventional chemotherapy. Shi et al. (30) reported that lenvatinib plus PD-1 inhibitors showed an active trend towards improving survival in advanced CCA patients after failure with cisplatin-gemcitabine chemotherapy. Similar trends have been found in advanced intrahepatic CCA patients treated with PD-1 inhibitors and lenvatinib after failure of chemotherapy (31). In a retrospective analysis of advanced CCA patients receiving lenvatinib plus PD-1/PD-L1 inhibitors and combined with gemcitabine and oxaliplatin (Gemox) chemotherapy, Zhu et al. (32) reported a median OS of 13.4 months, median PFS of 9.27 months, and ORR, DCR, and clinical benefit rate of 43.9%, 91.2%, and 73.7%, respectively. Shi et al. (33) analyzed advanced intrahepatic CCA patients treated with the same treatment regimen and reported median OS, PFS, and duration of response (DoR) of 22.5, 10.2, and 11.0 months, respectively. Thus, the combination of lenvatinib, PD-1/PD-L1 inhibitors, and Gemox chemotherapy may be an effective and tolerable treatment option for advanced CCA. Moreover, a multicenter retrospective study confirmed that early HAIC combined with PD-1 inhibitors could effectively prolong the OS of advanced CCA patients (34).

Because each treatment targets different pathways involved in tumor growth and survival, combining HAIC, lenvatinib, and PD-1 inhibitors may produce a synergistic anti-tumor effect. Additionally, this combination can overcome the resistance mechanisms of single therapies and enhance anti-tumor immunity. Therefore, HAIC plus lenvatinib with PD-1 inhibitors is a promising combination approach for treating malignancies. This combination has been extensively studied in liver cancer (19–21). Mei et al. (19) found that additional HAIC treatment was significantly associated with better treatment response and survival benefits in advanced hepatocellular carcinoma (HCC) patients treated with lenvatinib plus PD-1 inhibitors. Lai et al. (20) demonstrated that FOLFOX-HAIC plus lenvatinib and PD-1 inhibitors showed encouraging anti-tumor activity in patients with high-risk advanced HCC. An et al. (21) compared the efficacy and safety of lenvatinib and PD-1 inhibitors with lenvatinib alone in advanced HCC refractory to HAIC and showed that this combination therapy of PD-1 inhibitors plus lenvatinib has promising survival benefits. In addition, a systematic review of 311 clinical trials demonstrated that HAIC plus lenvatinib and PD-1 inhibitors effectively delayed disease progression, prolonged survival, and improved quality of life in HCC patients with portal vein tumor thrombus (35).

This study is the first to compare the clinical outcomes of combined treatment of HAIC plus lenvatinib with or without PD-1 inhibitors in advanced CCA patients, and has demonstrated the promising safety and efficacy of the combination of the three treatments. Our efforts are aimed at guiding future research and improving the quality and safety of management for advanced CCA in clinical practice. For advanced CCA patients who refuse systemic chemotherapy or who have failed previous chemotherapy or symptomatic support, we recommend that they receive HAIC plus lenvatinib with PD-1 inhibitors. Therefore, the results of this study may play a crucial role in the current management of advanced CCA.

There were some limitations to this study. Firstly, its retrospective nature confined the analysis to previously aggregated data. The study was susceptible to extraneous factors that were beyond our control, such as variations in data collection and probable partiality. Secondly, due to the limited number of patients included in the study, some of the significant findings may have been observed merely by chance. Therefore, the findings of this study should be confirmed in larger, prospective, cohort studies before being definitively accepted. Thirdly, the study sample was only composed of elderly Chinese patients, limiting our generalizability to other races undergoing similar treatment. Therefore, further study is required to verify if the observed trends are applicable to other countries.

## Conclusion

HAIC plus lenvatinib with PD-1 inhibitors is safe and well-tolerated in advanced CCA, and has the potential to prolong the survival of patients with advanced CCA. The addition of PD-1 inhibitors may enhance the efficacy of HAIC and lenvatinib. Therefore, the combined therapy has the potential to become a treatment option for advanced CCA. However, further study involving larger cohorts is necessary.

## Data availability statement

The original contributions presented in the study are included in the article. Further inquiries can be directed to the corresponding authors.

## Ethics statement

The studies involving human participants were reviewed and approved by the institutional review board and ethics committee at Beijing Tsinghua Changgung Hospital. The patients/participants provided their written informed consent to participate in this study.

## Author contributions

This work was designed by ZW, YJW, and YZ. The data were collected by YJW, BW, YL, YQW, ZR, and XY. The data were analyzed by ZW, YJW, and BW. This manuscript was written by ZW and YJW. QC provided assistance in the revision of the manuscript. All authors approved the final version.
